# An independent evaluation in a CRC patient cohort of microbiome 16S rRNA sequence analysis methods: OTU clustering, DADA2, and Deblur

**DOI:** 10.3389/fmicb.2023.1178744

**Published:** 2023-07-25

**Authors:** Guang Liu, Tong Li, Xiaoyan Zhu, Xuanping Zhang, Jiayin Wang

**Affiliations:** ^1^School of Electronic and Information Engineering, Xi'an Jiaotong University, Xi'an, China; ^2^Guangdong Hongyuan Pukong Medical Technology Co., Ltd., Guangzhou, China; ^3^School of Bioscience and Bioengineering, South China University of Technology, Guangzhou, China

**Keywords:** CRC, gut microbiome, denoising algorithms, comparison, DADA2, Deblur, OTU clustering

## Abstract

16S rRNA is the universal gene of microbes, and it is often used as a target gene to obtain profiles of microbial communities via next-generation sequencing (NGS) technology. Traditionally, sequences are clustered into operational taxonomic units (OTUs) at a 97% threshold based on the taxonomic standard using 16S rRNA, and methods for the reduction of sequencing errors are bypassed, which may lead to false classification units. Several denoising algorithms have been published to solve this problem, such as DADA2 and Deblur, which can correct sequencing errors at single-nucleotide resolution by generating amplicon sequence variants (ASVs). As high-resolution ASVs are becoming more popular than OTUs and only one analysis method is usually selected in a particular study, there is a need for a thorough comparison of OTU clustering and denoising pipelines. In this study, three of the most widely used 16S rRNA methods (two denoising algorithms, DADA2 and Deblur, along with *de novo* OTU clustering) were thoroughly compared using 16S rRNA amplification sequencing data generated from 358 clinical stool samples from the Colorectal Cancer (CRC) Screening Cohort. Our findings indicated that all approaches led to similar taxonomic profiles (with *P* > 0.05 in PERMNAOVA and *P* <0.001 in the Mantel test), although the number of ASVs/OTUs and the alpha-diversity indices varied considerably. Despite considerable differences in disease-related markers identified, disease-related analysis showed that all methods could result in similar conclusions. *Fusobacterium, Streptococcus, Peptostreptococcus, Parvimonas, Gemella*, and *Haemophilus* were identified by all three methods as enriched in the CRC group, while *Roseburia, Faecalibacterium, Butyricicoccus*, and *Blautia* were identified by all three methods as enriched in the healthy group. In addition, disease-diagnostic models generated using machine learning algorithms based on the data from these different methods all achieved good diagnostic efficiency (AUC: 0.87–0.89), with the model based on DADA2 producing the highest AUC (0.8944 and 0.8907 in the training set and test set, respectively). However, there was no significant difference in performance between the models (*P* >0.05). In conclusion, this study demonstrates that DADA2, Deblur, and *de novo* OTU clustering display similar power levels in taxa assignment and can produce similar conclusions in the case of the CRC cohort.

## Introduction

Colorectal cancer (CRC) is the third most common cancer globally, causing more than one million deaths annually (Brenner et al., 2014; Stoffel and Murphy, 2020). The occurrence and development of colorectal cancer often involve an adenoma-carcinoma process, which often occurs over many years and involves a variety of mechanisms and gene mutations (Fearon and Vogelstein, 1990; Jones et al., 2008). The early symptoms of CRC are not obvious: they consist of body discomfort, dyspepsia, occult blood in the stool, and other symptoms (Brenner et al., 2014). As the disease progresses, clearer symptoms gradually appear, including changes in defecation habits, blood in the stool, diarrhea, alternating diarrhea and constipation, and local abdominal pain, among others (Brenner et al., 2014). Not only does CRC inflict mental and physical distress on patients, but it also imposes a heavy economic burden and places pressure on patients and their families. Developed countries have now made significant advancements in CRC screening, resulting in a gradual decline in incidence (Brenner et al., 2014). Therefore, early cancer screening and timely intervention are important for CRC patients (Díaz-Tasende, 2018; Shaukat et al., 2021; Xi and Xu, 2021).

An increasing number of studies have shown a strong correlation between health and the intestinal microbiota; the microbiota can influence human health by regulating host immunity, inflammation, and cognitive function (Ashktorab et al., 2017; Zhang et al., 2017; Dalal et al., 2021). Studies have confirmed that the intestinal microbiota is a key environmental factor in the occurrence and development of CRC, as the composition of the intestinal microbiota is significantly different in CRC compared to that of healthy people (Zhang et al., 2017; Park et al., 2021). For example, an increase in *Fusobacterium nucleatum* has been confirmed to be closely related to CRC (Castellarin et al., 2012; Bullman et al., 2017). Therefore, fecal metagenomics may play a beneficial role in early screening and clinical diagnosis of colorectal cancer.

16S rRNA has a unique structure that contains both conserved and variable regions, and it is present in all known bacteria and archaea, so it is commonly used as a marker gene for bacterial community research using next-generation sequencing (NGS) technology (Coenye and Vandamme, 2003; Sanschagrin and Yergeau, 2014; Yarza et al., 2014; Muthappa et al., 2022). Not only can the 16S rRNA sequencing approach decrease the high cost of metagenomic sequencing, but it can also mitigate the problem of host contamination (Boers et al., 2019). However, sequencing errors can also introduce some non-real nucleotide differences (Kunin et al., 2010; Aird et al., 2011; Schloss et al., 2011). Traditionally, sequences are clustered into operational taxonomic units (OTUs) with a particular identity threshold (usually 97%) to reduce the interference of sequencing errors using the OTU clustering method (Edgar, 2013; Patin et al., 2013), and *de novo* clustering methods have been regarded as the optimal method of assigning the 16S rRNA gene to OTUs (Westcott and Schloss, 2015). In recent years, several denoising algorithms have been created to solve this problem, such as DADA2 (Callahan et al., 2016) and Deblur (Amir et al., 2017), which can correct sequencing errors by generating amplicon sequence variants (ASVs). DADA2 has been reported to be more accurate than the OTU clustering method in mock communities, as it can accurately resolve sequence variants differing by a single nucleotide and present in as few as two reads, identify more real variants, and output fewer spurious sequences; this provides alternative methods to explore strain-level variation (Callahan et al., 2016). In another study, it has been reported that both Deblur and DADA2 have high consistency, and both of them achieve outputs close to the ground truth in simulated communities (Amir et al., 2017). Deblur also exhibited higher performance stability than DADA2, with a higher frequency cutoff, when samples from the American Gut Project (http://americangut.org) underwent two sequencing runs, as a larger fraction of ASVs could be recalled in the second sequencing run (Amir et al., 2017).

Along with the widespread use of 16S rRNA sequencing, high-resolution ASVs have become more popular than OTUs. As only a single analysis method is usually selected in a particular study, there is a need for a thorough comparison of OTU clustering and denoising pipelines, as different methods may lead to different conclusions in some cases. However, there are not many studies on this topic. OTU clustering approaches have been compared using samples from chicken cecum (Allali et al., 2017) and preterm infants (Plummer and Twin, 2015), and a comparison of denoising algorithms using environmental samples was reported in 2018 (Nearing et al., 2018). However, a thorough comparison between denoising algorithms and OTU clustering approaches has yet to be conducted using clinical samples, and no comparison has been conducted specifically in colorectal cancer patients. In this study, based on 358 16S rRNA sequencing samples, including 184 CRC samples and 174 healthy human samples, a comparison was conducted between two selected denoising methods (DADA2 and Deblur), as well as *de novo* OTU clustering; additionally, disease-related markers were identified and the potential efficiency of a disease-diagnostic model based on machine learning algorithms was evaluated. The aim of this study was to assess whether similar biological conclusions regarding microbiome composition could be obtained using different methods.

## Methods

### Data acquisition and study design

**Figure 1:**
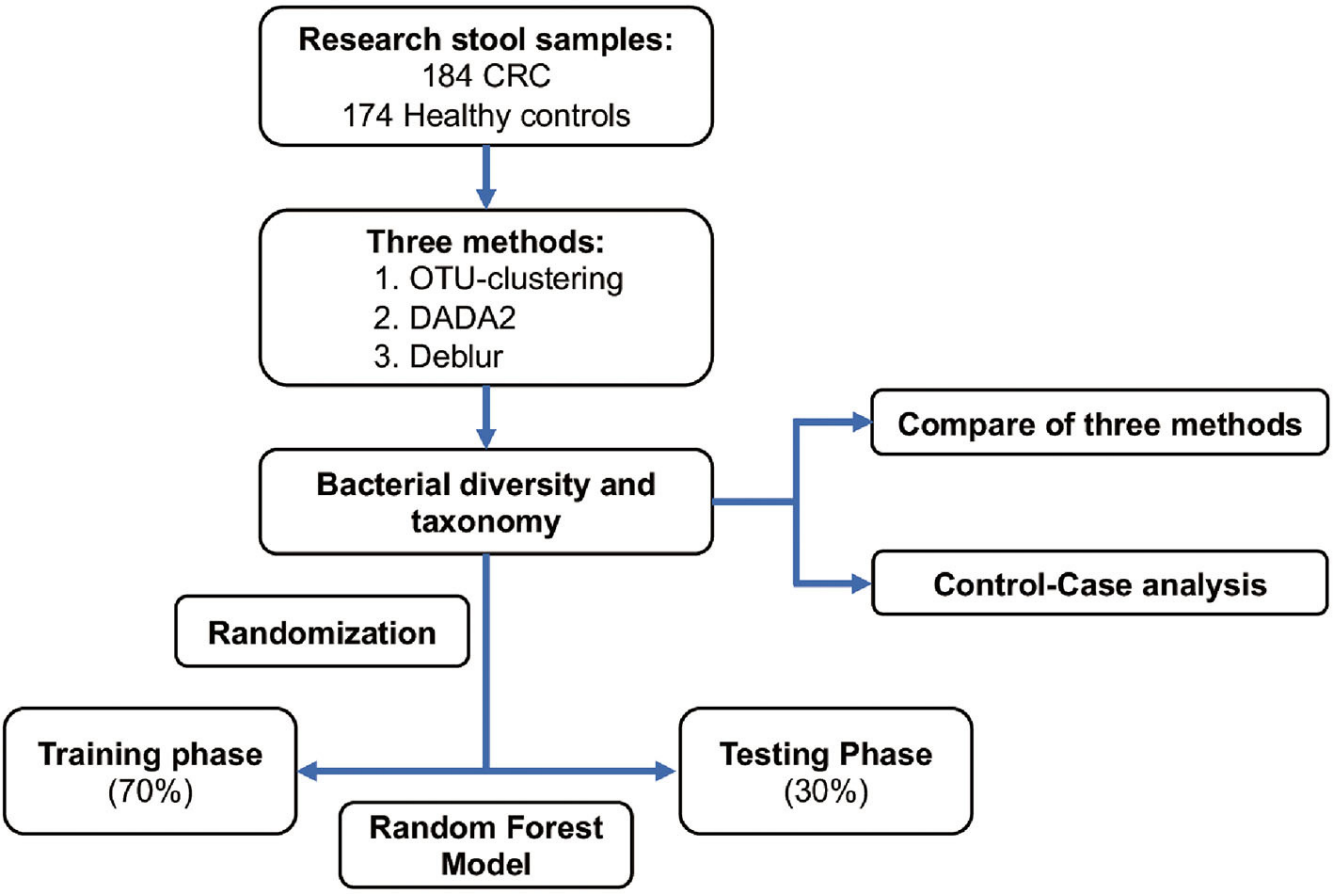
Study design and flow chart. A total of 358 stool samples from China were included in this study (184 samples from CRC patients and 174 from healthy controls). The fecal microbiota was assessed using 16S rRNA gene sequencing. Samples were randomly divided into a training set (70%) and a test set (30%). CRC, colorectal cancer; OTU clustering, *de novo* OTU-clustering-based analysis; DADA2,: DADA2 algorithm-based analysis; Deblur, Deblur algorithm-based analysis.

A total of 358 samples from a Chinese cohort were selected for inclusion in this study; these consisted of 184 CRC samples and 174 healthy control samples. For each of these samples, the V3-V4 region of the 16S rRNA gene was amplified using 319F/806R primer, and an Illumina MiSeq was used to generate 2 × 300bp reads. Reads were downloaded from the SRA database with the accession number PRJNA763023 (Yang et al., 2021). We selected samples from the older adult population (late-onset CRC patients and age-matched healthy controls) within the Fudan cohort, and selected samples with > 20,000 sequences in order to reduce the effect of lower numbers of sequences. The taxonomic composition of bacteria as established using multiple approaches (OTU clustering, DADA2, and Deblur) was first compared, and disease-related markers obtained based on the aforementioned methods were subsequently also compared (Figure 1). Next, the samples were randomly divided into a training set (70% of the data) for construction of a CRC classifier and a random forest model test set (30% of the data), which was used in a testing phase to verify the potential of the model.

### Data analysis

The 16S rRNA gene sequencing data were analyzed using the QIIME2 platform (v2020.2) (Hall and Beiko, 2018), which includes the VSEARCH software (Rognes et al., 2016) and software tools for DADA2 (Callahan et al., 2016) and Deblur (Amir et al., 2017). For the OTU clustering method: in brief, primers were removed using the Cutadapt plugin, and paired reads were merged using the “join-pairs” function of the VSEARCH plugin. The merged reads were then dereplicated (using the “dereplicate-sequences” function), singletons were filtered (using the “feature-table filter-features” function), chimeras were filtered (using the “uchime-ref” function, with the Greengenes13_8 97% OTU database as a reference), and the results were clustered at 97% identity using the *de novo* clustering method (the “cluster-features-de-novo” function) via the QIIME2 VSEARCH plugin with default settings. For DADA2-based methods, reads were truncated to lengths of 290 bp and 220 bp for the forward and reverse reads, respectively, to remove low-quality bases at the end of the reads, and the DADA2 plugin was run with default settings to construct the ASV feature table. For Deblur-based methods, the joined reads from VSEARCH were input into the Deblur plugin to construct the ASV feature table with default settings, and singletons were filtered. The OTUs and ASVs were then compared against the Silva Database (v138.1, https://www.arb-silva.de, download code: qiime2 rescript get-silva-data–pversion “138.1”–p-target “SSURef_NR99”) (Pruesse et al., 2007) using the “classify-sklearn” algorithm via the feature-classifier plugin (Bokulich et al., 2018). Data on read numbers are listed in Supplementary Table S1, and rarefaction plots are presented in Supplementary Figure S1. We also conducted alpha and beta diversity analyses using the diversity plugin in QIIME2.

### Bacterial taxonomic analysis

Typically, 16S rRNA data are examined at the genus level in further analysis, so genus profiles were entered into the following analyses. Bacterial taxonomic analyses were carried out, and comparisons between the three methods were conducted using the Wilcoxon rank sum test (Bauer, 1972). Linear discriminant analysis effect size (LEfSe, http://huttenhower.sph.harvard.edu/lefse/) (Segata et al., 2011) was used to identify disease-associated taxonomic features that could be used to explain differences between controls and cases. These features were selected via LEfSe analysis using the Kruskal–Wallis rank sum test (*P* < 0.05), and linear discriminant analysis (LDA score > 2) was used to assess the effect size associated with each feature.

### Analysis of diagnostic models

In order to differentiate CRC samples from healthy samples, a random forest (RF) model (Liu and Zhao, 2017; Yachida et al., 2019) was built using the random Forest package (v4.6) in R. Receiver operating characteristic (ROC) curves were constructed, and the area under the curve (AUC) was calculated to evaluate the diagnostic performance of these RF models using the pROC package (v1.17.0.1) in R. Subsequently, differences between the three models (constructed based on each of the three methods) in terms of diagnostic model efficiency were evaluated using the roc.test() function in the pROC package.

### Statistical analysis

The Mann–Whitney *U* test was employed to evaluate differences between groups. Permutational multivariate analysis of variance (PERMANOVA) was conducted to analyze the variance of the data generated using different methods, and the Mantel test was used to analyze the associations between these data. Spearman correlation analysis was performed to analyze the correlations between microbiota features. Plots were constructed using the ggplot2 package (v3.3.3) in R.

## Results

### Variation in taxonomic community composition across different methods

**Figure 2:**
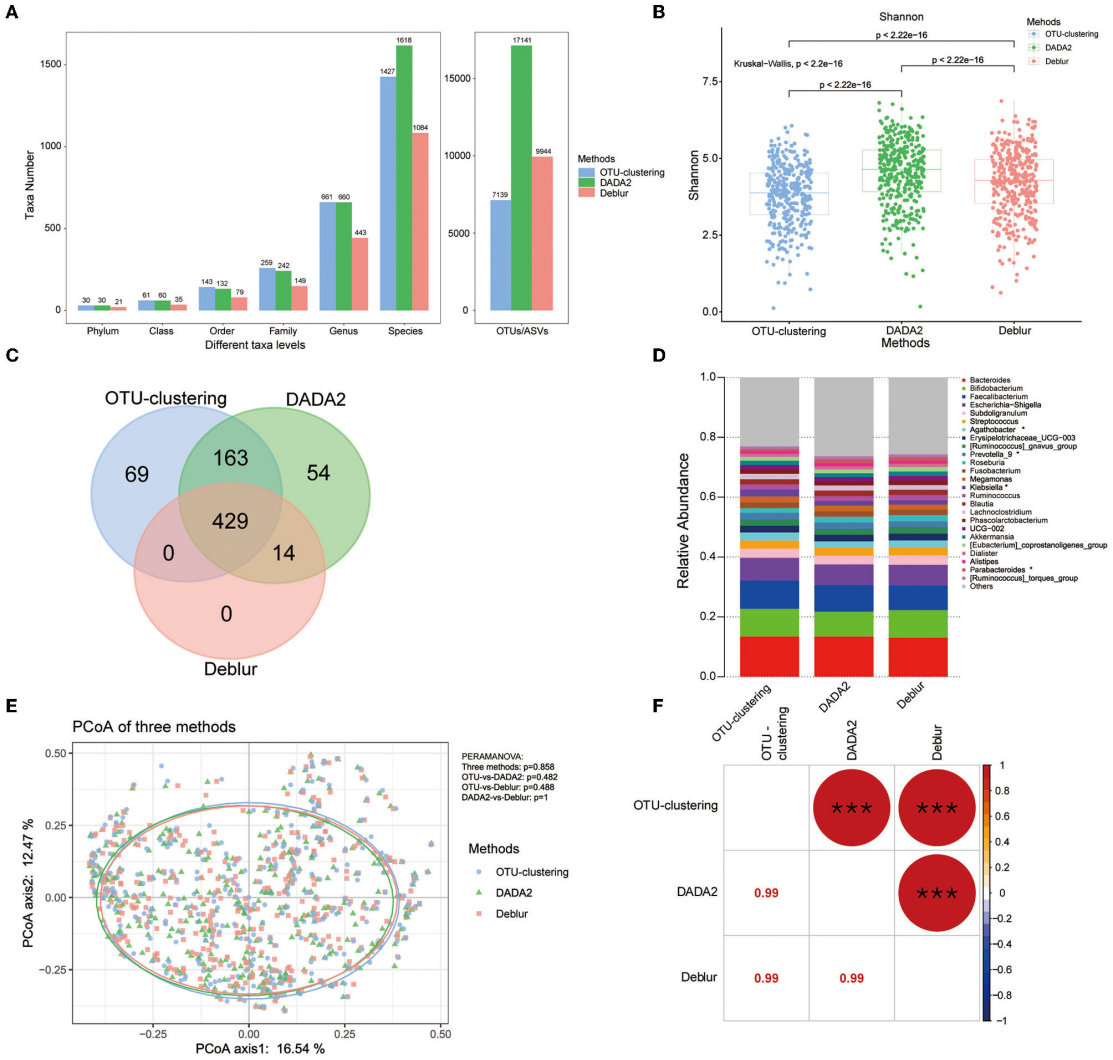
Comparison of bacterial diversity and taxonomic analyses under each of the three methods. **(A)** Number of taxa assessed at different taxonomic levels. **(B)** Shannon index across all samples calculated under each method. *P*-values for comparisons between two methods were calculated using the Mann–Whitney *U* test; all three methods were compared via the Kruskal–Wallis test. **(C)** Venn diagram showing analysis at the genus level. **(D)** Relative abundance of the top 25 genera across all samples; ^*^indicates Kruskal–Wallis *P*_*fdr*_ < 0.05. **(E)** PCoA analysis among the three methods, with *P*-values as calculated by PERMANOVA. **(F)** Mantel test comparing the taxonomic profiles obtained using each of the three methods. The number in the lower cell represents the Spearman correlation coefficient; the circle in the upper cell represents the *P*-value of the correlation. ^*^P < 0.05, ^**^P < 0.01, ^***^P < 0.001.

The number of OTUs/ASVs obtained using the three methods varied considerably, with DADA2 obtaining the most variants, followed by Deblur and OTU clustering (Figure 2A). At the taxonomic levels of genus and species, there was little difference between DADA2 and Deblur. However, OTU clustering obtained the largest number at the genus level and Deblur the smallest. In terms of alpha diversity, we found that all indices differed significantly between methods, as DADA2 produced the highest Shannon index, while OTU clustering produced the highest observed OTUs index and the highest Chao1 index (Figure 2B, Supplementary Figures S2A, B).

For exploration of the difference among the three methods in terms of taxonomic profiles generated, the genus level was selected for analysis. First, the Venn analysis indicated that a total of 429 genera could be detected by all three of the methods, accounting for 58.9% of the total number (the total number of detected genera was 729), and only 71 genera showed significant differences (Kruskal–Wallis, *P*_*fdr*_ < 0.05) in abundance among the three methods (Figure 2C, Supplementary Tables S2, S3). There were 163 genera that were identified by both OTU clustering and DADA2, but not Deblur, and there were significant difference between these two groups for 4 genera (Mann–Whitney test, *P*_*fdr*_ < 0.05) (Figure 2C, Supplementary Tables S2, S4). The genera obtained by OTU clustering and DADA2 mostly overlapped, with 592 shared genera, accounting for 81.2% of the total number. Regarding the 14 genera shared only by DADA2 and Deblur (and not OTU clustering), there was no difference between the two groups (Figure 2C, Supplementary Table S2). Finally, an interesting finding was that OTU clustering and Deblur did not identify any genera in common beyond the 429 identified by all methods (Figure 2C), indicating that the genera detected by Deblur were also detected by DADA2.

In most studies, taxa with higher abundance are more easily identified. Therefore, we also focused on comparison of the top 25 genera in terms of abundance; all these genera were detected by all three methods, with only four genera showing inter-method differences (Kruskal–Wallis, *P*_*fdr*_ < 0.05) (Figure 2D, Supplementary Table S6).

PCoA analysis based on Bray–Curtis dissimilarity was used to examine the sample clustering based on different methods; the results showed that the three methods could not be clearly distinguished, as most samples were clustered together, with a *P* > 0.05 for PERMANOVA (Figure 2E). In addition, a Mantel test was performed to evaluate the correlations among the taxonomic profiles obtained by the three methods; the results showed that there were significant correlations among the three profiles (*r* = 0.99, *P* < 0.001) (Figure 2F). These results indicated that, although there were some differences in the number of taxa obtained by the three methods, the taxonomic profiles obtained using the different methods were strongly correlated, and the relative abundances of the expected taxa under each method were strikingly similar.

### Analysis of case–control differences

**Figure 3:**
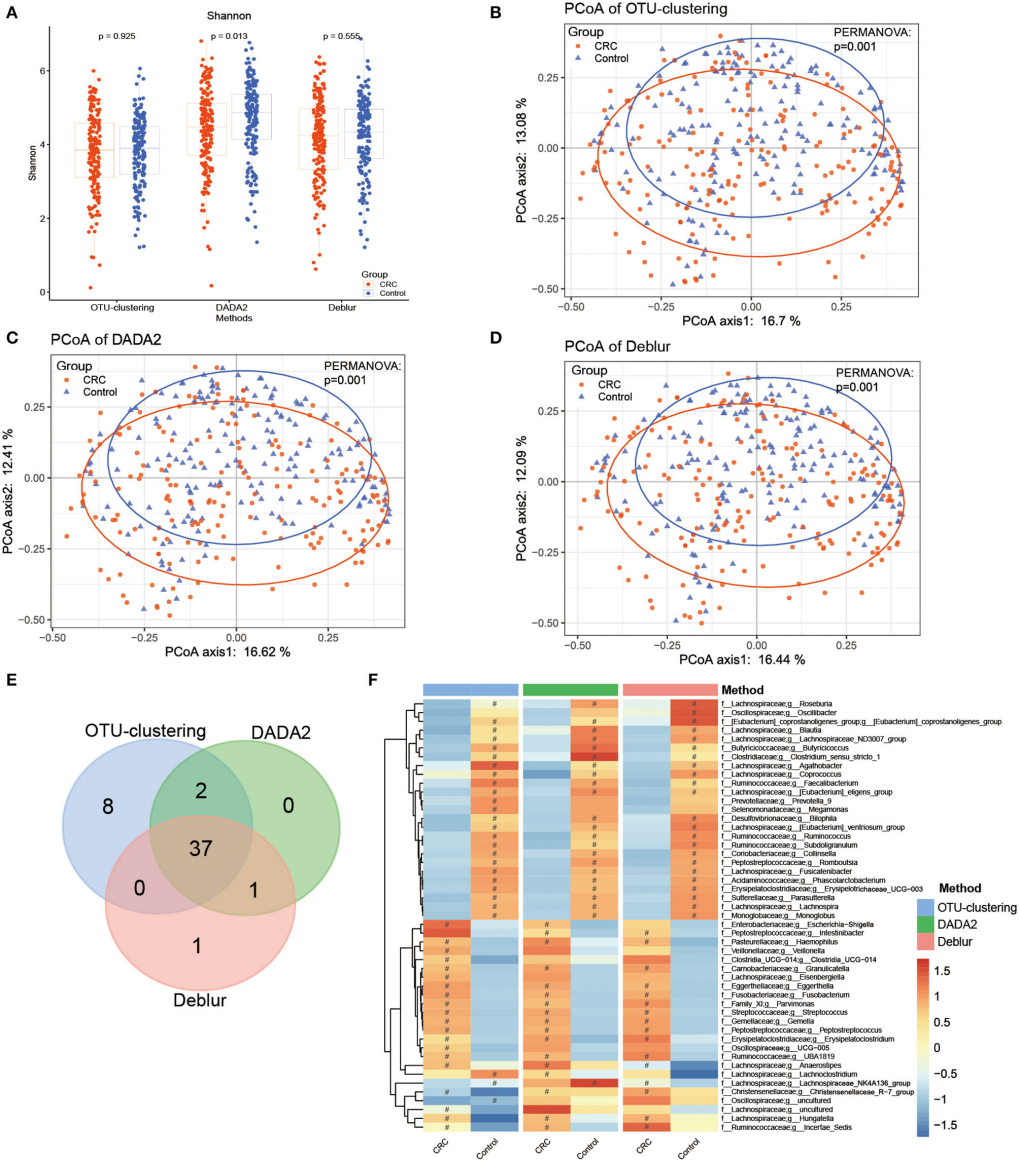
Bacterial diversity and taxonomic analysis of differences between cases and controls. **(A)** Comparison of Shannon index between cases and controls, with *P*-values as calculated for the Mann–Whitney U test. **(B–D)** PCoA analysis comparing cases and controls under **(B)** the OTU clustering method, **(C)** the DADA2 method, **(D)** the Deblur method, with *P*-values calculated by PERMANOVA. **(E)** Venn diagram showing analysis of disease-related markers based on LEfSe analysis. **(F)** Heatmap of disease-related markers based on LEfSe analysis. ^#^represents enriched groups.

We analyzed the differences between cases and controls to evaluate whether the different algorithms produced different disease-related outcomes. First, we found that although the Shannon index was lower for CRC patient samples than for healthy samples across all three methods, only DADA2 identified a significant difference between the groups (Figure 3A). In contrast, inter-group differences on the Chao1 index and the observed OTUs index were observed under all methods (Supplementary Figures S2C, D).

According to the results of PCoA analysis based on Bray–Curtis dissimilarity (Figures 3B–D), the samples from the CRC patients and healthy controls could be clearly distinguished into two groups; the *P*-values of PERMANOVA were also significant, indicating that the different methods could produce the same conclusion.

Subsequently, LEfSe analysis was conducted to identify disease-related markers that distinguished the CRC group and the healthy group. Under this analysis, OTU clustering produced the largest number of markers (47 markers), followed by DADA2 (40 markers) and Deblur (39 markers) (Figures 3E, F, Supplementary Table S7, Supplementary Figures S3A–C). A total of 49 markers were obtained, among which 37 could be detected using all three methods (Figures 3E, F). All three methods indicated enrichment of 13 genera in the CRC group; these included *Fusobacterium, Gemella, Peptostreptococcus*, and *Streptococcus*, which have been reported on widely in CRC research (Kwong et al., 2018; Brennan and Garrett, 2019; Wong et al., 2019). In contrast, the different methods identified 23 genera as enriched in the healthy group; these included *Roseburia, Faecalibacterium*, and *Blautia*, which have been proven to have a positive effect on human health. However, the results for *Lachnoclostridium, Escherichia-Shigella*, and *Megamonas* differed between the three methods. This indicates that, though there were a small number of differences in disease-related markers, identification of most of the markers could be reproduced using different methods.

### Differences between disease-diagnostic models

**Figure 4:**
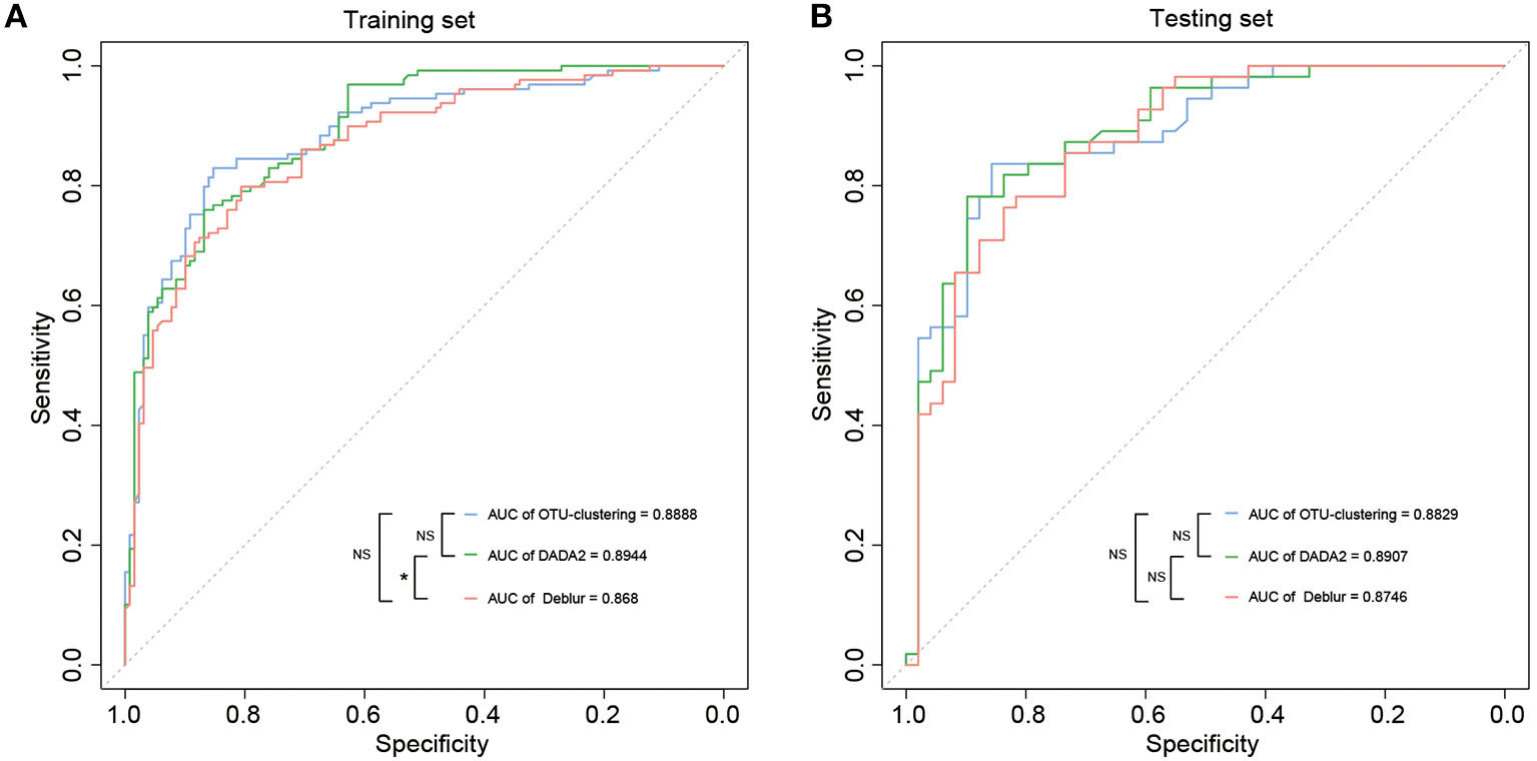
Disease classification models based on disease-associated taxa. **(A, B)** The AUC value of disease classification models using the random forest in the training set **(A)** and testing set **(B)** based on disease-associated taxa from LEfSe. The color of the lines means the data were obtained from different methods, and “*” suggested significant differences (*P* < 0.05) between models using the roc.test() function, while NS suggested that there was no significant difference (*P* > 0.05).

One of the scenarios for application of an understanding of the gut microbiome in CRC is early screening and auxiliary diagnosis; this is also the most valuable application of studies of this type. Therefore, we used the random forest algorithm to construct disease diagnosis models based on the disease-related markers identified through LEfSe analysis (Supplementary Table S7), and evaluated the diagnostic efficiency of these models on the basis of the AUC according to the associated ROC curves. All models could distinguish between CRC and healthy samples well, as the AUC values were >86%. The results indicated that the model based on DADA2 analysis exhibited the best performance (training set: AUC = 89.4%, CI 85.72–93.16%; test set: AUC = 89.7%, CI 82.71–95.44%), followed by the OTU clustering model (training set: AUC = 88.9%, CI 84.83–92.93%; test set: AUC = 88.3%, CI 81.72–94.87%) and finally the Deblur model (training set: AUC = 86.8%, CI 82.45–91.15%; test set: AUC = 87.5%, CI 80.68–94.23%) (Figures 4A, B, Supplementary Figures S4A–F). However, the only significant difference in AUC was between the DADA2 and Deblur models in the training set (*P* < 0.05) (Figure 4A). These results indicated that there was no significant difference between the three methods in terms of the efficiency of the resulting diagnostic model.

## Discussion

We compared the results obtained by using three methods (DADA2, Deblur, and OTU clustering) to analyze data from a clinical cohort. First, we found that DADA2 and Deblur could identify more ASVs than OTUs in the community (Figure 2A). The reason for this is that, during the error-correction process of the denoising algorithms, the identity standard was 100%, and the obtained ASVs were considered to represent real individuals. In contrast, during the process of *de novo* OTU clustering, the identity standard was 97%, without no reliance on any database, and the number of OTUs was relatively low. The results under the DADA2 and Deblur methods indicated higher alpha diversity (Figure 2B, Supplementary Figures S2A, B). Comparing the two denoising methods, the number of ASVs obtained using DADA2 was much higher than that obtained using Deblur (Figure 2A), which may be because only singletons were removed from all samples by default under DADA2; in contrast, under Deblur, not only were singletons in a single sample removed, but sequences below 10 in all samples were also removed. These results suggest that DADA2 may be more sensitive to rare species. A report by Nearing et al. has also claimed that DADA2 can obtain more ASVs than Deblur in analysis of simulated and environmental microbial communities (Nearing et al., 2018).

All three methods included a process for removal of singletons with low frequencies, which not only removed some errors but also discarded some rare taxa. This requires users to make a reasonable selection of thresholds according to the purpose of their analysis. OTU clustering has clear disadvantages in this regard, as some sequences below the threshold cannot be accurately distinguished. The classic *de novo* clustering algorithm is Uparse (Edgar, 2013), whose developer, Robert Edgar, argued that the 97% threshold for taxonomic classification was too low; he proposed that the threshold for 16S sequences with full length should be 99% and that the threshold for the V4 region should be 100% in order to improve accuracy (Edgar, 2018). However, use of a higher threshold in OTU clustering may introduce identification of spurious taxonomic units without correction of sequencing errors, as employed under DADA2 and Deblur. In addition, data from studies based on OTU clustering cannot be combined for further analysis, which introduces major challenges in drawing comparisons between different studies, while DADA2 and Deblur do not have this problem.

After obtaining feature sequences (ASVs/OTUs), we compared them with the reference database containing known taxa in order to obtain taxonomic information using the classify-sklearn algorithm, which is a form of naive Bayes classifier (a classification method based on machine learning) (Bokulich et al., 2018). Previous studies have shown that the classify-sklearn algorithm could provide more accurate annotation information at the genus and species levels (Kaehler et al., 2019; Ziemski et al., 2021). The results showed that, as the annotation level increased, the gaps in the number of taxa obtained using each of the three methods gradually narrowed, especially the gap between DADA2 and OTU clustering (Figure 2A). At the species level, the largest number of taxa was obtained using DADA2 (1,618), followed by OTU clustering (1,427) and then Deblur (1,084). At the genus level and higher, the numbers obtained using DADA2 and OTU clustering were very similar, whereas Deblur consistently identified the smallest number of taxa, with a loss of approximately 30%−70%. The number of taxa identified using Deblur was the lowest at the species level and higher, which may be related to the process used under Deblur for filtering sequences below 10 in all samples, resulting in the removal of low-frequency taxa. DADA2 removed only singletons in all samples, which not only increased the number of ASVs but also made it easier to obtain more taxa, especially at the species level. However, DADA2 and OTU obtained similar numbers of taxa at the genus level and higher, indicating that DADA2 had this advantage only at the species level. Therefore, DADA2 is recommended in cases where the researchers need more information on specifically on ASVs and at the species level.

Usually, further analysis of 16S rRNA data is conducted at the genus level, so the genera profiles were selected for subsequent analyses. Venn diagram analysis showed that the three methods obtained a total of 729 genera, of which 429 genera (58.9%) could be identified using all three methods (Figure 2C). Among these genera, 71 differed significantly among methods (Kruskal–Wallis *P*_*fdr*_ < 0.05) (Figure 2C). OTU clustering and DADA2 shared 81% of the detected genera, while DADA2 and Deblur shared only 60%. Regarding the 71 genera with inter-method differences, most of the genera were enriched under OTU clustering (Supplementary Table S3), possibly because OTU clustering retained more sequences. Although the number of genera identified using DADA2 and OTU clustering was similar, each also identified some unique genera that was not identified by the other, and the relative abundance of these unique genera was very low: the maximum abundance was 3.454e-06 among the 69 genera identified only by OUT clustering and 4.18e-06 among the 69 genera identified identified only by DADA2 (Figure 2C, Supplementary Table S5). Traditionally, genera with higher abundance as regarded as playing an important role in the community. These results showed that the top 25 genera could be detected by all three methods, including four significant taxa (Kruskal–Wallis, *P*_*fdr*_ < 0.05, Supplementary Table S6). This indicates, although the three methods will lead to identification of different genera, the differences are mainly reflected in genera with low relative abundance, and researchers need to choose appropriate methods according to their objectives.

PCoA analysis showed that the samples could not be distinguished well through use of different methods. PERMANOVA also indicated that the use of different methods had no significant effect on the microbial profile obtained (*P*>0.05) (Figure 2E). The microbial profiles were robustly correlated across methods (Mantel test, *n* = 358, *r* = 0.99, *P* < 0.001) (Figure 2F), confirming the strong similarity of the results across the three methods.

In clinical research, disease-related bacteria are important for researchers. Therefore, we compared the results of case–control analyses based on each of the three methods. First, in alpha diversity analyses, inter-group differences in Shannon index were observed only under DADA2, while inter-group differences on the Chao1 index and the observed OTUs index were observed under all methods, with the CRC group showing significantly lower diversity than the healthy group (Figure 3A, Supplementary Figures S2C, D). This result was consistent with those of previous CRC studies, which have shown that alpha diversity is reduced in the CRC population (Yang et al., 2021); other studies have also observed no significant difference in the Shannon index (Feng et al., 2015; Wu et al., 2021). This finding suggests that researchers must select the appropriate alpha index according to their methods. PCoA analysis showed that all three methods could distinguish successfully between the CRC group and the healthy group, with *P* < 0.05 in PERMANOVA (Figures 3B–D), confirming the difference between the cases and controls as compared using different methods.

LEfSe analysis was used to explore disease-related microbial markers. A total of 49 markers (OTU-clustering: 47; DADA2: 40; Deblur: 39) were obtained using the three methods, of which 37 markers (75.5%) could be reproduced using a different method; the enrichment trend was consistent for most markers (Figure 3F, Supplementary Figures S3A–C). For example, *Haemophilus, Granulicatella, Eggerthella, Fusobacterium, Parvimonas, Streptococcus, Gemella, Peptostreptococcus*, and other genera (for a total of 13) were found under all three methods to be enriched in the CRC group. *Fusobacterium*, especially *Fusobacterium nucleatum*, is an important marker of CRC (Kostic et al., 2012; Yang et al., 2021) and an opportunistic pathogen in many chronic oral and intestinal diseases, such as inflammatory bowel disease (IBD) (Weng et al., 2019). *Fusobacterium nucleatum* has been reported to promote glycolysis and oncogenesis in CRC by upregulating the lncRNA ENO1-IT1 (Hong et al., 2021) and promoting CRC cell migration by modulating the long non-coding RNAs keratin7-antisense (KRT7-AS) and keratin7 (KRT7) (Chen et al., 2020). *Streptococcus* is also a common pathogenic genus that often causes inflammation and bacteremia, possibly promoting CRC (Kwong et al., 2018; McAuliffe et al., 2019). *Peptostreptococcus* is an anaerobic, gram-positive bacterium, and *Peptostreptococcus anaerobius* has been reported to promote CRC and modulate tumor immunity (Long et al., 2019). *Haemophilus* is an opportunistic pathogen that may cause hemorrhagia and acute meningitis. Finally, *Parvimonas* is a fastidious, anaerobic, gram-positive coccus that is widely found among healthy human oral and gastrointestinal flora, and previous studies have demonstrated that *Parvimonas micra* is associated with CRC (Löwenmark et al., 2020; Xu et al., 2020).

*Roseburia, Faecalibacterium, [Eubacterium]_eligens_group, Bilophila, Phascolarctobacterium, Butyricicoccus, Blautia*, and other genera (for a total of 23) were found to be enriched in the healthy group. *Butyricicoccus* and *Faecalibacterium* are butyric acid producers, which play an important role in intestinal and host health and act as protectors against CRC (Miquel et al., 2013; Zhou et al., 2018; Chang et al., 2020). Butyric acid is the main energy source of colonic epithelial cells and can reduce the pH in the colon, regulate human immunity, and exert anti-inflammatory effects. *Faecalibacterium* has been found in multiple studies to be significantly decreased in many diseases (Lopez-Siles et al., 2017), including Crohn's disease (CD) (Martinez-Medina et al., 2006), ulcerative colitis (UC) (Machiels et al., 2014), inflammatory bowel diseases (IBD) (Frank et al., 2007), and CRC. *Roseburia* can ferment various carbohydrates and may play a positive role in exerting anti-inflammatory effects and preventing CRC (Machiels et al., 2014). *Phascolarctobacterium* produces short-chain fatty acids (SCFAs) and plays various important roles in maintaining human health, such as enhancing gastrointestinal function, reducing inflammation levels, and influencing metabolic state and mood of the host (Wu et al., 2017). Finally, *Blautia* produces acetic acid, which not only contributes to gas emissions in the intestine but also exerts anti-inflammatory effects (Liu et al., 2021; Miyake et al., 2021).

The markers obtained using each of the three methods were not always completely consistent. For example, *Lachnoclostridium* was found to be enriched in the healthy group under OTU clustering, enriched in the CRC group under DADA2, and not significantly enriched in either group under Deblur. *Lachnoclostridium* has been reported to be enriched in colorectal adenoma and cancer (Li et al., 2020). *Escherichia-Shigella* was found to be enriched in CRC under DADA2 and OTU clustering, which is consistent with previous studies (Wang et al., 2012; Han et al., 2019). *Intestinibacter* was found to be enriched in CRC under DADA2 and Deblur; this genus has been reported to be associated with immune-mediated inflammatory diseases (IMIDs) and to be found in greater abundance in CD (Forbes et al., 2018). *Megamonas* was found to be enriched in the healthy group only under OTU clustering. *Megamonas* has previously been reported to be enriched in healthy controls compared with cachectic cancer patients (Ubachs et al., 2021). *Megamonas* can ferment many carbohydrates, producing various intestinal epithelial cell nutrients, such as acetic acid, propionic acid, and lactic acid (Tian et al., 2020; Ubachs et al., 2021). *Oscillibacter* was found to be enriched in the healthy group only under Deblur; this genus has previously been proven to be enriched in normal tissue samples compared to their respective tumor counterparts (Loke et al., 2018). These results indicate that, even though a small number of markers differed between the methods, most of the disease-related markers identified were consistent across methods.

Finally, random forest (RF) models were constructed to evaluate diagnostic efficiency based on each of the three methods. For the training set, the highest AUC was obtained for the DADA2 model, at 89.4%, followed by 88.9% for the OTU clustering model and 86.8% for the Deblur model; the only significant difference in AUC was between DADA2 and Deblur (*P* < 0.05) (Figure 4A, Supplementary Figures S4A, C, E). This trend was subsequently verified in the test set (DADA2 > OTU clustering > Deblur), but there were no significant differences among the AUCs for the three models (*P* > 0.05) (Figure 4B, Supplementary Figures S4B, D, F). These results indicate that each of the different methods can achieve good diagnostic efficiency, with DADA2 being the best.

In conclusion, although there were differences in the number of OTUs/ASVs obtained using the three methods, the differences in the numbers of taxa were smaller, especially for the comparison between DADA2 and OTU clustering at the genus level. Moreover, the microbial profiles were strongly correlated. This indicates that the results obtained using the three methods are comparable. Case–control analysis also showed that the three methods could yield similar results, with mostly consistent identification of CRC-related markers. However, it should also be noted that the three methods were performed with the default parameters, and adjusting some of the parameters in the selected method could help users to obtain their desired results.

## Data availability statement

The original contributions presented in the study are included in the article/Supplementary material, further inquiries can be directed to the corresponding author.

## Author contributions

JW and GL designed the research study. GL and TL performed bioinformatics analysis and wrote the manuscript. XZha and XZhu edited the manuscript. All authors read and approved the final manuscript.

## Supplementary material

Supplementary Figure S1 Rarefaction plots of the Shannon index **(A–D)** and observed OTUs index **(E–H)**.

Supplementary Figure S2 **(A, B)** Chao1 index **(A)** and observed OTUs index **(B)** of all samples using three methods. *P*-values comparing two methods were calculated using the Mann–Whitney U test; the Kruskal–Wallis test was used in the three-method comparison. **(C, D)** Chao1 index **(C)** and observed OTUs **(D)** for cases and controls based on three methods. *P*-values for comparisons of the two groups were calculated using the Mann–Whitney *U* test.

Supplementary Figure S3 LEfSe analysis comparing CRC patients and controls based on three methods.

Supplementary Figure S4 The performance of the disease classification models based on the three methods was evaluated using AUCs. OTU clustering training set **(A)** and test set **(B)**; DADA2 training set **(C)** and test set **(D)**; Deblur training set **(E)** and test set **(F)**. The confidence interval for the AUC value was calculated.

Supplementary Table S1 The reads were obtained per sample.

Supplementary Table S2 The number of genera from venn analysis among three methods.

Supplementary Table S3 Kruskal–Wallis analysis of genera detected by three methods.

Supplementary Table S4 Mann-Whitney test of genera detected by OTU-clustering and DADA2 alone in venn analysis.

Supplementary Table S5 The averege relative abundance of genera only detected by OTU-clustering or DADA2.

Supplementary Table S6 Kruskal–Wallis analysis of top25 genera detected by three methods.

Supplementary Table S7 LEfSe analysis between CRC and Control based on three methods.
