# Supplementary figures and images for: An independent evaluation in a CRC patient cohort of microbiome 16S rRNA sequence analysis methods: OTU clustering, DADA2, and Deblur
Source: Front Microbiol. 2023 Jul 25;14:1178744. doi: 10.3389/fmicb.2023.1178744 (PMC10408458; doi:10.3389/fmicb.2023.1178744)

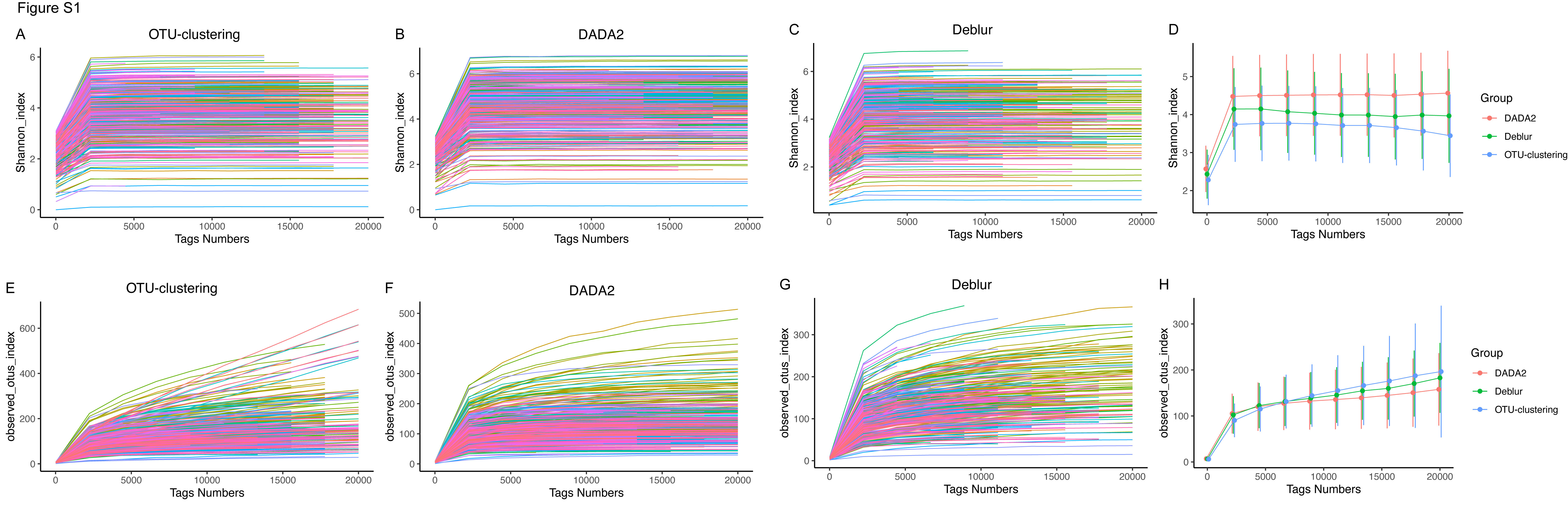

Supplement: Supplementary Figure S1 — Rarefaction plots of the Shannon index (A–D) and observed OTUs index (E–H). [file Image_1.jpeg]

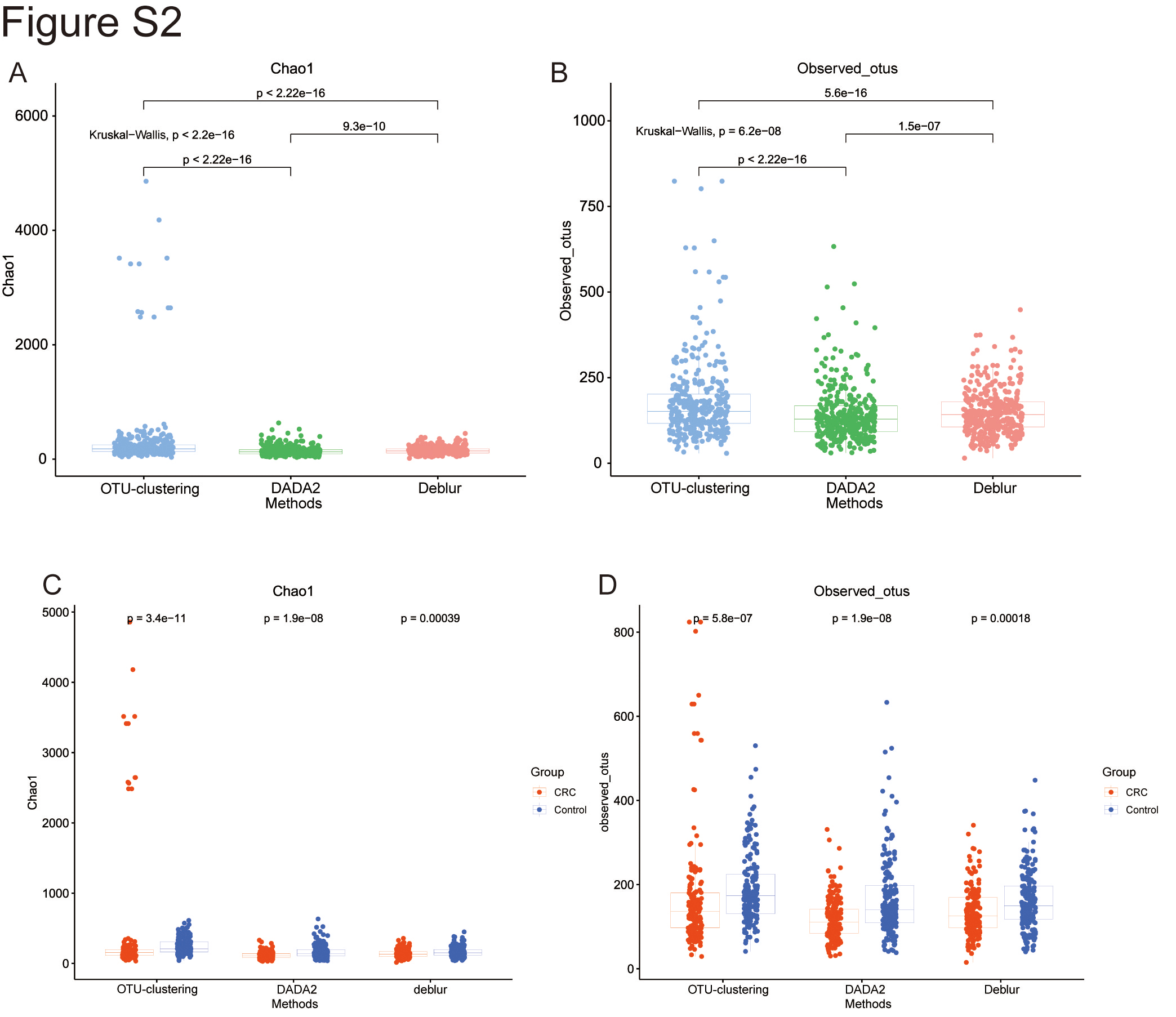

Supplement: Supplementary Figure S2 — (A, B) Chao1 index (A) and observed OTUs index (B) of all samples using three methods. P-values comparing two methods were calculated using the Mann–Whitney U test; the Kruskal–Wallis test was used in the three-method comparison. (C, D) Chao1 index (C) and observed OTUs (D) for cases and controls based on three methods. P-values for comparisons of the two groups were calculated using the Mann–Whitney U test. [file Image_2.jpeg]

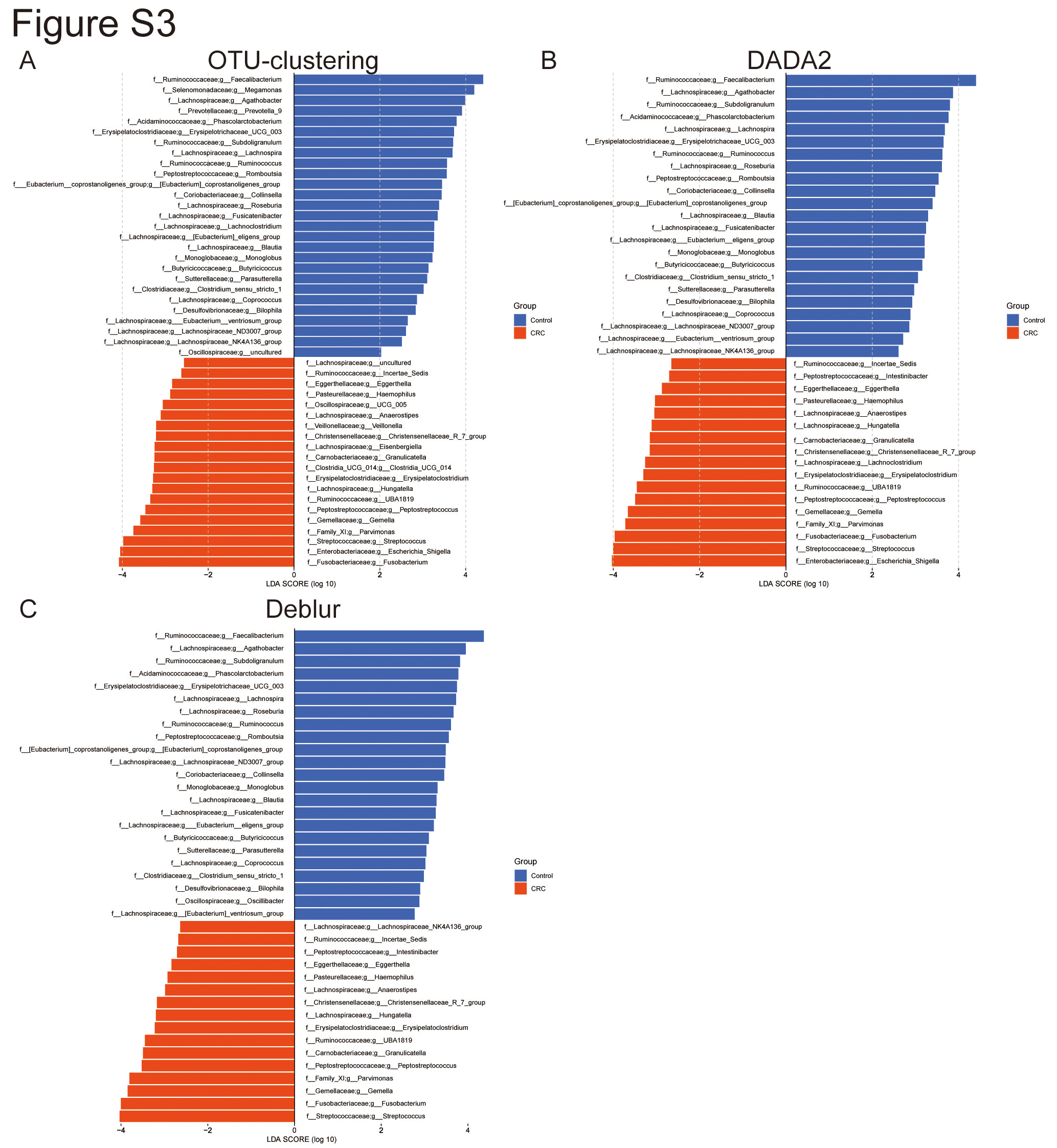

Supplement: Supplementary Figure S3 — LEfSe analysis comparing CRC patients and controls based on three methods. [file Image_3.jpeg]

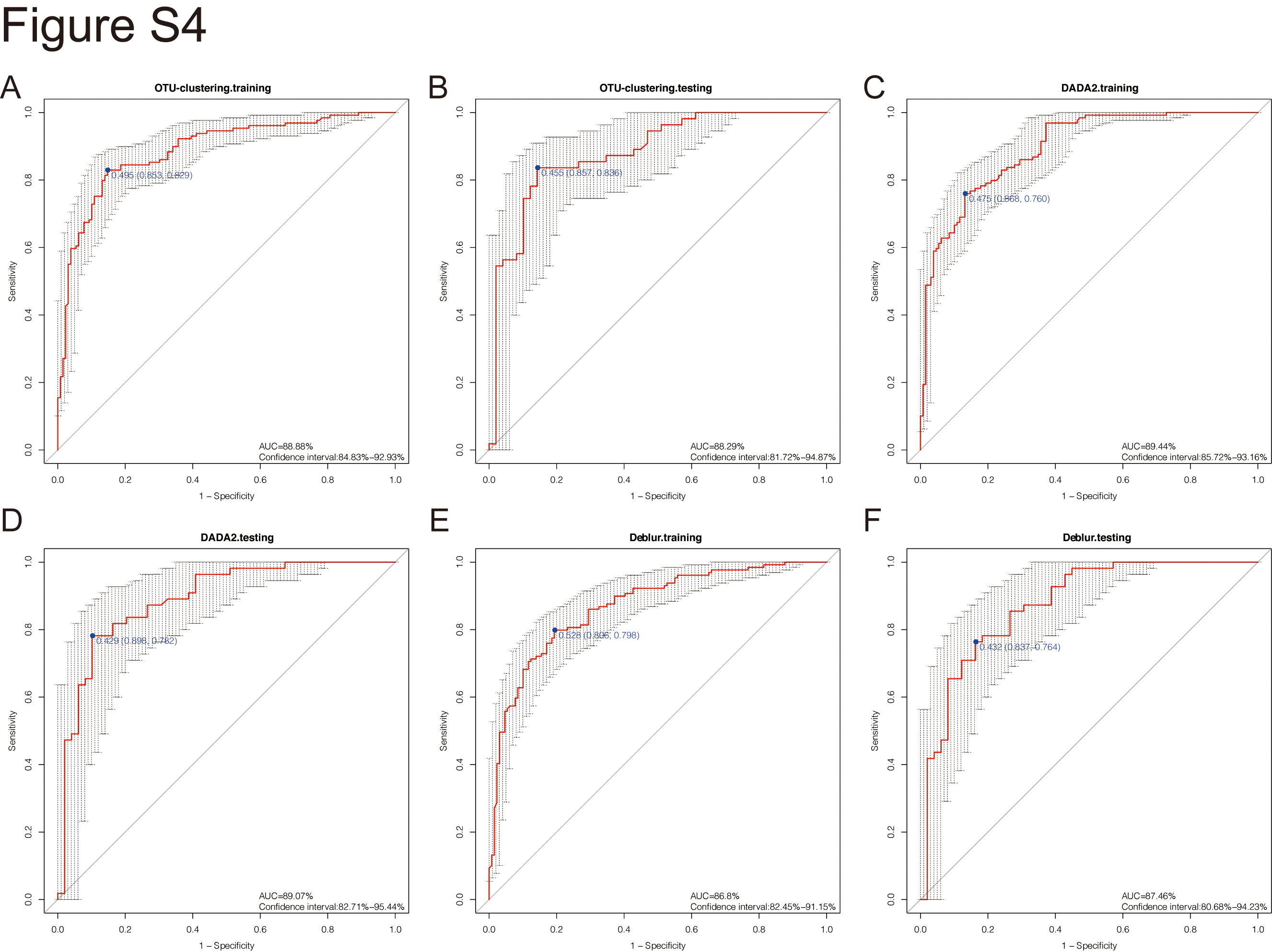

Supplement: Supplementary Figure S4 — The performance of the disease classification models based on the three methods was evaluated using AUCs. OTU clustering training set (A) and test set (B); DADA2 training set (C) and test set (D); Deblur training set (E) and test set (F). The confidence interval for the AUC value was calculated. [file Image_4.jpg]
